# Effects of backpacking holidays in Australia on alcohol, tobacco and drug use of UK residents

**DOI:** 10.1186/1471-2458-7-1

**Published:** 2007-01-02

**Authors:** Mark A Bellis, Karen E Hughes, Paul Dillon, Jan Copeland, Peter Gates

**Affiliations:** 1 Centre for Public Health, Liverpool John Moores University, Castle House, North Street, Liverpool, L3 2AY, UK; 2 National Drug and Alcohol Research Centre, University of New South Wales, Sydney, NSW 2052, Australia

## Abstract

**Background:**

Whilst alcohol and drug use among young people is known to escalate during short holidays and working breaks in international nightlife resorts, little empirical data are available on the impact of longer backpacking holidays on substance use. Here we examine changes in alcohol, tobacco and drug use when UK residents go backpacking in Australia.

**Methods:**

Matched information on alcohol and drug use in Australia and the UK was collected through a cross sectional cohort study of 1008 UK nationals aged 18–35 years, holidaying in Sydney or Cairns, Australia, during 2005.

**Results:**

The use of alcohol and other drugs by UK backpackers visiting Australia was common with use of illicit drugs being substantially higher than in peers of the same age in their home country. Individuals showed a significant increase in frequency of alcohol consumption in Australia compared to their behaviour in the UK with the proportion drinking five or more times per week rising from 20.7% (UK) to 40.3% (Australia). Relatively few individuals were recruited into drug use in Australia (3.0%, cannabis; 2.7% ecstasy; 0.7%, methamphetamine). However, over half of the sample (55.0%) used at least one illicit drug when backpacking. Risk factors for illicit drug use while backpacking were being regular club goers, being male, Sydney based, travelling without a partner or spouse, having been in Australia more than four weeks, Australia being the only destination on their vacation and drinking or smoking five or more days a week.

**Conclusion:**

As countries actively seek to attract more international backpacker tourists, interventions must be developed that target this population's risk behaviours. Developing messages on drunkenness and other drug use specifically for backpackers could help minimise their health risks directly (e.g. adverse drug reactions) and indirectly (e.g. accidents and violence) as well as negative impacts on the host country.

## Background

Young people's behaviour changes when holidaying away from their usual residence [1-3]. Typically on short one or two week holidays, risk taking behaviours (in particular alcohol and drug use) escalate as constraints from education or work commitments reduce, social mores relax and opportunities to consume substances (through for example lower cost and increased availability) increase [2-5]. Such escalation is linked to increases in unsafe sex[6], accidents (including road traffic accidents [7,8]) and possible damage to mental health through excessive consumption of alcohol and drugs [9-11]. Furthermore, the holiday environment of indulgence and excess can lead to social norms and peer pressure encouraging individuals who have never used illicit drugs to begin consumption [1,2,12]. While use of drugs anywhere can have adverse reactions including overdose, anxiety, panic attack and dehydration, use abroad is often more dangerous because of unknown supplies, lack of knowledge of local health services, and often isolation from usual community support [13].

Increasingly, for the young residents of many countries such international travel is no longer the exception [14] but almost an expectation on an annual basis. From the UK alone over four million young people aged 18–30 are estimated to travel abroad every year[15,16]. Most such journeys are holidays taken to European destinations with each vacation lasting one or two weeks[15,17]. However, a large and increasing proportion of young people also set aside time for longer journeys (frequently a year) often including stays in multiple countries before returning home[18,19]. Typically backpacking describes tourists on such protracted holidays who carry their necessary belongings in a backpack[20]. In contrast to shorter international holidays, backpacking often includes living on a low budget with less indulgent expenditure and can also involve individuals seeking employment while abroad, especially in distant countries such as Australia and New Zealand [19-21]. During the five year period from 1999 to 2003 the number of domestic and international backpackers travelling in Australia grew 25% (753,000 to 943,000); accounting for 11% of overseas visitors[22]. The largest single source of backpackers visiting Australia was the UK, accounting for 27% of individuals (121,500 [23]).

To date, studies on risk behaviour and youth travel have focused largely on short-term vacations. In particular those on substance use among backpackers have been predominantly qualitative [24,25], providing some evidence of experimentation with drugs abroad yet little epidemiological information on this type of youth travel and its relationship with substance use[12]. However, there are many reasons why behaviour changes associated with backpacking might differ from those during shorter trips to holiday resorts (e.g. Ibiza[2]). Popular backpacker destinations, such as Sydney, Australia, are general tourist destinations not specifically designed for youth tourism but catering for a wider holidaying and endemic population. Consequently, regulations regarding drug use or drunkenness may be more stringently enforced [26] and opportunities to purchase illicit substances more restricted. Further, with money often more limited on such longer vacations, finance may also be a restrictive factor and the necessity to work while travelling may require individuals to avoid late nights, drug use and excessive alcohol consumption for working parts of each week[27]. Equally however, participating in employment may increase opportunities to meet local people and obtain access to local illicit drug markets.

With levels of young people backpacking increasing, health professionals (in travellers' home countries and those they visit) require information on how backpacking may alter patterns of substance use, what health risks such changes represent[11] and what interventions should be targeted at these populations. To address such issues, here we compare the alcohol, tobacco and drug using behaviour of 1008 UK residents in the last 12 months before they leave the UK and when backpacking in Australia.

## Methods

As one of the most popular international destinations for backpackers from the UK[23,28], Australia was chosen as the country in which to conduct the survey. A cross sectional cohort study design was chosen which utilised a single questionnaire to measure individuals' backpacking experience (e.g. length of trip, destinations visited), behaviour in Australia and, for within individual comparisons, their behaviour in the UK prior to their trip[29]. Questions addressed alcohol, tobacco, illicit drug use and sexual behaviour and had been previously validated and utilised in studies of short holidays within Europe[2,3]. However, some questions were adapted to examine the longer lengths of stay routinely experienced by backpackers in Australia and to include substances more commonly used in that country (e.g. methamphetamine[30]). Consequently, the range of illicit drugs examined included cannabis, ecstasy, amphetamine (i.e. amphetamine sulphate), methamphetamine, crystal meth or ice (methamphetamine in crystalline form), cocaine, crack, lysergic acid diethylamide (LSD), ketamine, gammahydroxybutrate (GHB), heroin and steroids.

Given the diversity of nightlife and other tourist environments in Australia, two contrasting locations were identified in which to sample backpackers. The first, Sydney (population approximately 4.2 million[31]), is a major international metropolis with a flourishing nightlife, busy international transport connections and consequently often the point of arrival and departure for backpackers visiting the country[21]. In contrast, around 2700 kilometres north is Cairns (population approximately 120,000[31]); the third most common tourist destination in the country after Sydney and Brisbane. Popular with overseas tourists and particularly international backpackers, the city has a less well developed nightlife but one that is very much designed for young travellers.

Ethics approval was received from the University of New South Wales Human Ethics Committee, the auspicing body for the study, and research methods complied with the Helsinki Declaration. Questionnaires were administered in Sydney (28^th ^April–22^nd ^November 2005) and Cairns (1^st^–6^th ^August 2005). In both locations backpacking hostels were utilised as the sites for sampling potential respondents and within each hostel researchers approached individuals on a convenience basis. Inclusion criteria for the survey were being age 18 to 35 years, having already been in Australia for at least two weeks and being a UK national. In all cases researchers explained to potential respondents the content of the survey and its anonymous nature. Informed consent was recorded and participants were reimbursed $AUS10 for their time. All individuals meeting the inclusion criteria and agreeing to participate (n = 1012 of 1114 approached; participation rate 90.8%) were given a questionnaire, pen and plain envelope in which to seal the completed questionnaire and return it to the researcher. Completed questionnaires were entered into SPSS (Statistical Package for Social Sciences) for analysis[32]. At this stage a further four questionnaires were excluded as responses did not meet survey inclusion criteria for age or period of stay (n = 3) and one questionnaire was spoilt (i.e. no effort had been made to complete questions and the questionnaire had been defaced).

Analysis utilised a combination of Chi Square, Mann Whitney U, McNemar and Wilcoxon signed rank tests with logistic regression being used to control for confounding relationships between variables when examining predictors of drug use. For logistic regression analyses, amphetamine, methamphetamine and crystal meth were combined into a single category of "used amphetamine type", and a category of "used any illicit drug" was also created.

## Results

Table 1 provides the basic demographics of respondents, details of their travel plans, whether they had to visit a doctor or hospital in Australia and how frequently they went to nightclubs in Australia. Overall the median age of the sample was 23 years with significantly more males being aged 25 to 35 years (Table 1). Most (79.3%) individuals were not travelling with a long-term partner or spouse. For almost 40% of individuals Australia was their first destination since leaving the UK and for a similar proportion it was to be their last destination before returning to the UK (Table 1). The median planned stay was 25 weeks but males planned to stay significantly longer than females (Table 1) with over a third expecting to stay more than 40 weeks. However, length of stay at time of interview was not significantly different between sexes (Table 1). The vast majority (97.3%) of backpackers went to nightclubs (i.e. clubbing) at least once a week (Table 1). Further, during their stay approximately one fifth of all backpackers had required hospital treatment or a visit to a doctor at least once. Over one in ten (10.9%) of those who required such attention stated it was alcohol-related and 6.5% that it was drug-related.

**Table 1:** General sample characteristics and comparison between sexes of UK backpackers in Australia

	All		Male		Female			
Survey area	n	%	n	%	n	%	*X*^2^	P
*Cairns*	259	25.7	156	23.4	103	30.3		
*Sydney*	749	74.3	511	76.6	237	69.7	5.62	<0.05
Age								
*18 to 20*	198	19.6	138	20.7	60	17.6		
*21 to 24*	457	45.3	283	42.4	174	51.2		
*25 to 35*	353	35.0	246	36.9	106	31.2	6.96	<0.05
Abroad with partner/spouse	205	20.7	122	18.6	83	24.9	5.44	<0.05
Australia first destination from UK	396	39.3	268	40.2	128	37.6	0.61	0.436
Australia final destination before UK	368	36.7	247	37.3	121	35.8	0.20	0.651
Australia only destination^1^	232	23.0	155	23.2	77	22.6	0.04	0.833
Stay to date (weeks)								
*2 to 4*	339	33.6	215	32.2	124	36.5		
*>4 to 12*	316	31.3	210	31.5	105	30.9		
*>12*	353	35.0	242	36.3	111	32.6	2.08	0.354
Stay remaining (weeks)								
*2 to 4*	278	28.3	155	23.9	123	36.9		
*>4 to 12*	273	27.8	182	28.1	91	27.3		
*>12*	431	43.9	311	48.0	119	35.7	20.74	<0.001
Total stay (weeks)								
*2 to 12*	302	30.8	178	27.5	124	37.2		
*>12 to 40*	359	36.6	239	36.9	120	36.0		
*>40*	321	32.7	231	35.6	89	26.7	12.23	<0.005
Visited hospital/doctor in Australia	202	20.3	122	18.5	80	23.8	3.86	<0.05
Nights clubbing per week								
*None*	26	2.7	15	2.4	11	3.5		
*1*	161	17.0	89	14.1	72	22.7		
*2 to 4*	635	67.1	435	69.2	199	62.8		
5 or more	125	13.2	90	14.3	35	11.0	12.97	<0.01

^1^Travelling from the UK to Australia and back without holiday periods in other countries on either route

Use of both licit and illicit substances by backpackers in Australia was common (Table 2). There was no significant change in proportions of individuals who drank in Australia compared with proportions who drank in the UK (Table 2). However, there was a significant change in frequency of consumption with 35% of alcohol-using backpackers increasing frequency of drinking in Australia, and the proportion drinking five or more days a week rising from 20.7% (UK) to 40.3% (Australia; *X*^2 ^= 91.31; P < 0.001).

**Table 2:** Levels of substance use in UK backpackers while in the UK and when visiting Australia

Frequency of use	Alcohol	Tobacco	Cannabis	Ecstasy	Cocaine	Crack	Amphetamine	Methamphetamine	Crystal Meth	LSD	GHB	Ketamine	Steroids	Heroin
**UK**	%	%	%	%	%	%	%	%	%	%	%	%	%	%
*Never used*	1.2	26.0	27.0	48.3	63.5	95.6	71.4	85.4	93.2	75.4	92.5	87.4	96.9	95.2
*Used but not in last 12 months*	1.1	15.1	20.1	17.8	13.9	3.3	16.4	9.6	4.6	19.0	6.0	8.9	2.2	3.5
*Less than 1 day a week*	6.8	7.5	25.6	23.8	18.8	0.8	8.8	3.1	1.6	5.1	1.4	3.3	0.6	0.8
*1 day a week*	16.8	4.1	7.9	6.1	2.0	0.2	1.8	1.1	0.2	0.2	0.0	0.1	0.1	0.0
*2 to 4 days a week*	53.5	9.2	9.6	3.5	1.4	0.0	1.0	0.5	0.3	0.2	0.0	0.1	0.0	0.2
*5 or more days a week*	20.7	38.1	9.7	0.5	0.4	0.1	0.7	0.3	0.1	0.2	0.1	0.2	0.2	0.2
**Australia**														
*Never used in Australia*	2.1	38.1	49.9	69.6	93.9	99.1	92.4	96.9	98.4	96.8	99.2	98.0	98.7	98.6
*Less than 1 day a week*	4.7	7.7	20.1	21.3	5.6	0.6	6.2	2.0	1.4	3.0	0.5	1.5	0.7	1.0
*1 day a week*	8.4	4.4	9.4	6.7	0.6	0.1	0.6	0.3	0.1	0.1	0.1	0.2	0.1	0.2
*2 to 4 days a week*	44.5	10.2	10.2	2.1	0.3	0.0	0.6	0.7	0.0	0.0	0.2	0.2	0.0	0.0
*5 or more days a week*	40.3	39.7	10.3	0.4	0.2	0.2	0.2	0.1	0.1	0.1	0.0	0.1	0.3	0.2
**Countries recently used in^1^**														
*Neither*	1.6	36.3	40.0	60.5	76.1	98.5	85.9	93.8	96.7	92.9	98.0	95.1	98.7	97.8
*UK Only*	0.5	1.8	9.9	9.1	17.3	0.6	6.6	3.1	1.7	3.9	1.3	2.9	0.2	0.8
*Australia Only*	0.7	4.9	7.1	5.6	1.3	0.4	1.9	1.2	1.1	1.4	0.5	1.2	0.4	0.9
*Both*	97.2	57.1	43.0	24.9	5.3	0.5	5.7	1.9	0.5	1.8	0.2	0.8	0.7	0.5
McNemars	0.77	<0.001	<0.05	<0.01	<0.001	0.75	<0.001	<0.01	0.34	<0.005	0.1	<0.05	0.69	1.00
**Change in frequency of use^2^**														
*Australia>UK*	35.0	13.2	28.4	9.2	5.7	0.0	7.0	10.5	20.0	5.6	0.0	0.0	28.6	0.0
*UK>Australia*	5.1	5.6	16.2	16.8	22.6	20.0	31.6	36.8	20.0	22.2	50.0	12.5	14.3	20.0
*Unchanged*	59.9	81.2	55.4	74.0	71.7	80.0	61.4	52.6	60.0	72.2	50.0	87.5	57.1	80.0
Wilcoxon's Signed Rank	<0.001	<0.001	<0.005	<0.01	<0.05	0.32	<0.005	0.08	0.66	0.13	0.32	0.32	0.41	0.32

^1^Users in the UK are those that consumed the drug in their last 12 months in the UK. For Australia they are those that consumed the drug at any time during their current visit.

^2^Calculated only for those individuals that have currently used (see above)^1 ^in both locations.

Cannabis was the most commonly used illicit drug both in the UK and in Australia. Over 40% of individuals used it in both the UK and Australia with a small but significant tendency for those who used in just one country to have used only in the UK (Table 2). However, over a quarter of those using in both countries used cannabis at higher frequencies in Australia while only 16.2% reduced frequency of consumption when backpacking. A quarter (24.9%) of individuals used ecstasy in both Australia and the UK, with a further 5.6% using in Australia although they had not used in the previous 12 months spent in the UK (Table 2). In fact, 2.7% (27/1005) of backpackers surveyed used ecstasy for the first time while on their current trip to Australia (cf. cannabis 3.0%; cocaine 0.6%; amphetamine 0.9%; methamphetamine 0.7%; crystal meth 0.7%). In contrast to cannabis, those using ecstasy in both countries were significantly more likely to use at a higher frequency in the UK (Table 2). Furthermore, the number of ecstasy tablets they consumed during a typical night of use was also significantly lower in Australia (median, inter quartile range; UK 2.5, 2.0–4.0, Australia 2.0, 1.0–2.0; Z = 9.13, P < 0.001). For cocaine, amphetamine, LSD, ketamine and even methamphetamine, those using in one country but not the other were significantly more likely to have used in the UK (Table 2). Further, for those using cocaine or amphetamine in both countries frequency of use was more likely to decrease than increase when backpacking.

Logistic regression analyses were employed to examine independent demographic and behavioural predictors of having used cannabis, ecstasy and cocaine as well as combined categories of "any illicit drug" and "amphetamine types" in Australia (see methods for category components). Use of drugs in the UK, although strongly related to use in Australia (Table 2), was not used as a predictive factor but variables were included that help identify groups at high risk of recreational drug use in Australia and could assist with targeting appropriate interventions. Being resident in Sydney (not Cairns) was a strong predictive factor for having used any illicit drug, cannabis and especially cocaine (adjusted odds ratio, 17.67; Table 3) and this may reflect easier access to drugs in Sydney. Being male, travelling without a long-term sexual partner and frequently visiting nightclubs all predicted any illicit drug use, and cannabis and ecstasy use. Length of stay completed at participation was positively related to these behaviours as well as use of cocaine. Further, daily (≥5 days/week) tobacco use was strongly associated with all types of other substance use including the category amphetamine types, while daily alcohol use (≥5 days/week) was associated with all categories except cocaine (Table 3).

**Table 3:** Logistic regression analysis of factors predicting use of illegal drug in Australia during their current backpacking vacation

		Used any illegal drug^1^			Cannabis			Ecstasy			Cocaine			Amphetamine Types^2^		
		AOR	(95%CI)	P^3^	AOR	(95%CI)	P	AOR	(95%CI)	P	AOR	(95%CI)	P	AOR	(95%CI)	P
Survey area	*Cairns*	Ref		**	Ref		**				Ref		**			
	*Sydney*	1.74	(1.21–2.51)		1.86	(1.27–2.72)					17.67	(2.33–134.12)				
Age	*18 to 20*				Ref		*									
	*21 to 24*				1.56	(1.05–2.33)	*									
	*25 to 35*				1.01	(0.66–1.54)	NS									
Sex	*Female*	Ref		***	Ref		***	Ref		**						
	*Male*	1.80	(1.31–2.46)		1.85	(1.35–2.55)		1.70	(1.2–2.41)							
Australia only destination^4^	*No*				Ref		**	Ref		*						
	*Yes*				1.64	(1.13–2.37)		1.49	(1.03–2.17)							
Abroad with partner/spouse	*Yes*	Ref		***	Ref		***	Ref		*						
	*No*	2.18	(1.5–3.16)		2.08	(1.42–3.03)		1.60	(1.05–2.42)							
Stay to date (weeks)	*2 to 4*	Ref		***			***	Ref		***	Ref		*			
	*>4 to 12*	1.77	(1.23–2.55)	**	1.63	(1.13–2.36)	**	2.33	(1.55–3.5)	***	1.56	(0.65–3.74)	NS			
	*>12*	2.34	(1.62–3.39)	***	2.42	(1.67–3.5)	***	3.10	(2.08–4.63)	***	3.08	(1.29–7.4)	*			
Total stay (weeks)	*2 to 12*										Ref		*			
	*>12 to 40*										0.43	(0.16–1.14)	NS			
	*>40*										0.95	(0.37–2.46)	NS			
Nights clubbing per week	*<= 1*	Ref		**	Ref		**	Ref		**						
	*2 to 4*	1.84	(1.24–2.72)	**	1.70	(1.14–2.54)	**	2.49	(1.51–4.11)	***						
	*5 or more*	2.85	(1.57–5.18)	**	2.58	(1.43–4.65)	**	2.79	(1.46–5.3)	**						
Daily alcohol-Australia^5^	*No*	Ref		*	Ref		**	Ref		*				Ref		*
	*Yes*	1.42	(1.01–2.01)		1.64	(1.17–2.31)		1.48	(1.05–2.1)					1.74	(1.1–2.75)	
Daily tobacco-Australia^5^	*No*	Ref		***	Ref		***	Ref		***	Ref		***	Ref		***
	*Yes*	3.24	(2.35–4.47)		2.92	(2.13–4.01)		2.80	(2.03–3.86)		3.29	(1.87–5.77)		2.37	(1.5–3.77)	

Stay remaining (weeks) and whether individuals had had to visit a hospital or doctor in Australia were included in the analysis but were not significant for any types of substance use. For all other independent variables relationships that were not significant are left blank. Reference categories are identified with 'Ref' and overall levels of significance for each independent variable are presented in the same row as the reference category. AOR = Adjusted Odds Ratio; CI = Confidence Intervals.

^1^Cannabis, Ecstasy, Amphetamine, Methamphetamine, Crystal Meth, Cocaine, Crack, LSD, Ketamine, GHB, Heroin and Steroids.

^2^Includes Amphetamine, Methamphetamine and Crystal Meth.

^3 ^Significance is shown as * P < 0.05, **P < 0.01, ***P < 0.001, NS = not significant.

^4^Travelling from the UK to Australia and back without holiday periods in other countries on either route.

^5 ^Here daily is defined as anyone reporting use five or more days a week.

**Figure 1:**
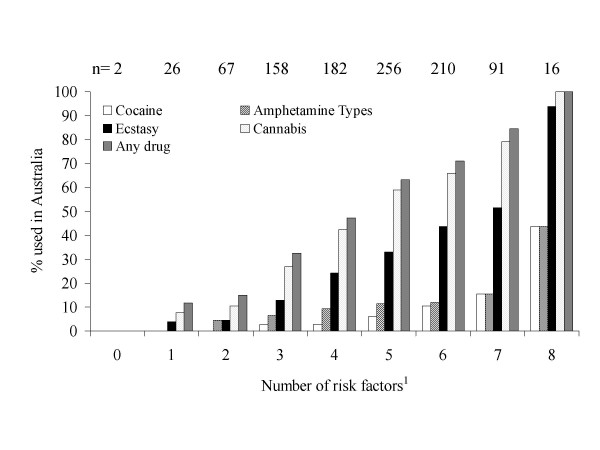
Relationship between number of drug use risk factors individuals display and consumption of illicit drugs in Australia. ^1^Risk factors were identified through logistic regression analysis (Table 3) and include all independent variables which were significant predictors of at least two dependents categories of drug use. Risks factors are residence in Sydney, male, only visiting Australia, traveling without a long term partner or spouse, stay to date over four weeks, night clubbing more than once a week and alcohol or tobacco consumption ≥5 days per week.

All independent variables from the logistic regression that were significant predictors of at least two dependents were categorised as risk factors for drug use while backpacking. Each respondent was scored according to how many of these risk factors they displayed (scale 0–8). Figure 1 shows the relationship between number of risk factors and proportions using different drug types. Thus, all individuals having a score of eight used cannabis in Australia, most used ecstasy (93.8%) and nearly half used amphetamine types (43.8%) or cocaine (43.8%).

## Discussion

Here, we compared substance use of UK backpackers while travelling in Australia with their behaviour in the UK. As sampling was on a convenience basis we could not identify how representative participants were of all UK backpackers travelling in Australia. However to minimise bias, participants were approached by researchers and refusal to participate occurred on only 9.2% of occasions. A second methodological concern was bias in recall of behaviour in the UK. However, the periods over which individuals were asked to recall substance use were not dissimilar to established surveys which routinely evaluate individuals' substance use in the past 12 months[33]. Finally, backpackers self assessed the types of drugs they used. This again is a not an unusual methodology for large drug surveys. However, whilst in Australia backpackers may have thought they used amphetamine (i.e. amphetamine sulphate or 'speed' in the UK) but may actually have used methamphetamine powder (also called 'speed' in Australia); which can look like amphetamine and can be used in the same way. Similarly, those reporting use of Ecstasy may again have unwittingly used methamphetamine, which is frequently found in 'pills' sold in Australia [34].

Across the general population of England and Wales ecstasy use in 16–34 year olds in the last year (2004/05[33]) was 3.9% compared with 33.9% amongst our respondents (age 18–35) in the 12 months before leaving the UK. Consequently, we conclude that UK backpackers are more likely to be drug users than average members of the British population of comparable age. As such, those choosing to go on backpacking holidays are a risk group for drug-related harms and consequently a target group for harm minimisation and prevention measures whether at home or abroad. Currently, although some information and advice on alcohol and drug use abroad is available to those intending to backpack abroad (e.g. through a gap year website[35]), once abroad interventions to address substance use amongst backpackers are limited.

Whilst in Australia, cannabis was by far the most commonly used illicit drug by backpackers. There, around 10% used ≥5 days a week, 30 individuals used for their first time and those who used in both countries used more frequently in Australia (Table 2). Qualitative studies of backpackers suggest that cannabis is seen as a 'safe' drug that allows the user to stay in control[25]. When travelling, sometimes alone, safety can be paramount[36] and taking tablets of unknown quality or other drugs with immediate and dramatic disorientating effects may be less attractive. Further, cannabis also plays a specific social role when it (usually in cigarette form) is shared between friends and to make new acquaintances[25]. Tobacco, another substance that can occupy a similar niche, also increased in both numbers using and frequency of use in Australia (Table 2). For most other drugs however backpackers were less likely to use in Australia. Thus, despite studies suggesting some uptake by travellers of local drug patterns[24], reported prevalence of methamphetamine use (the second most popular illicit drug among Australians[37]) by backpackers was actually lower than in the UK.

For all drugs, individuals who used in both countries were most likely to use at the same frequency in each (Table 2). However, for all drugs except cannabis and steroids, those changing frequency of use were significantly more likely to use at a lower frequency in Australia (Table 3). These are very different changes in drug using behaviour from those seen on short holidays to nightlife resorts (e.g. Ibiza[2]) where changes in drug use are typified by drug bingeing with significant increases in frequency of use. The length of time spent overseas, financial resources and even having to work are all likely to play a part in limiting drug use on backpacking vacations. However, access to drugs may also be a major factor. Here, use in Australia of "any illicit drug" and specifically cannabis, ecstasy and cocaine were all related to length of stay to date. This is consistent with individuals requiring time to source drugs and develop confidence to buy and consume them in a country where drug laws are routinely enforced[38]. The same constraints appear to have also limited initiation of individuals into drug use or of existing drug users into the use of drugs more commonly found in Australia. Thus, cocaine use amongst backpackers in Australia was more prevalent than methamphetamine despite this being more commonly used in Australian nightlife and cheaper to purchase than cocaine[39]. In fact, only seven individuals who had never tried methamphetamine in the UK reported having done so in Australia with the same number also trying crystal meth in Australia for the first time. However, backpackers may have inadvertently used methamphetamine assuming, for example, it was amphetamine. Although we cannot measure the size of this effect, here 7.6% of backpackers reported using amphetamine during their stay despite, in Australia, most amphetamine being forms of methamphetamine[40]. Importantly however, methamphetamine has stronger stimulant effects, is linked to physical and mental health problems (e.g. seizures, paranoia, depression), violence and risky sexual behaviour [41,42] and should individuals later realise what they have used they may seek this stronger drug for future use.

Overall despite relatively low levels of recruitment into drug use, there were still a substantial number of individuals who used illicit drugs in Australia (55.0% used an illicit drug at least once when backpacking). Such individuals are more likely to visit nightclubs more than once a week, be male, Sydney based, travelling without their partner or spouse, have been in Australia over four weeks, be visiting Australia only and also be daily or near daily (≥5 days a week) drinkers and smokers. Such profiling should help information to be specifically developed for, and then directed at, those leaving the UK to backpack while also allowing services in Australia to target further information and advice at these groups. Tailored approaches to prevention and harm minimisation have been shown to be more effective than general information provision that ignores the specific characteristics of the target groups[43]. Thus, information linking drugs with alcohol and tobacco would be highly applicable to this group. However, frequent alcohol use by backpackers is not only a predictor of drug use in Australia but may also be a substantial concern in its own right. Backpackers show significant increases in frequency of alcohol use when in Australia which contrasts strongly with the protective role backpacking plays against use of most illicit drugs. Numbers drinking ≥5 days a week nearly doubled from 20.7% in the UK to 40.3% in Australia. While this study did not measure the amount of alcohol consumed each night, this increase in use over the long time periods associated with backpacking is a cause for concern and intervention. Alcohol is not only associated with direct acute (e.g. poisoning) and long term (e.g. liver disease) effects on health but also plays a major role in accidents (including deaths of drunk pedestrians), violence and engagement in unprotected sex [6,44]. In hot climates it can also play a role in over exposure to the sun (e.g. through sleeping on the beach and sitting outside bars[45]), thus increasing risks of skin cancer[46]. Further, drunkenness as well as drug use and dealing can adversely affect host populations while related events such as a fatality amongst visitors can devastate a tourist industry[47]. Consequently, health interventions targeting backpackers in Australia are likely to be of benefit not only to travellers but also to local populations.

## Conclusion

As increasing numbers of individuals spend more time abroad, their health protection and promotion requirements need to be more adequately understood and addressed. With a high probability of UK backpackers being illicit drug users, and many increasing their alcohol consumption while abroad, backpacking holidays represent an important opportunity to deliver drug and alcohol related messages to those most at risk. Such messages should stress that most backpackers reduce illicit drug use while travelling. Of course, health issues will always arise for some individuals as part of lengthy holidays abroad. However, the potential benefits of backpacking appear to include not only a thriving tourist industry for host countries and a growing market for travel operators but also valued personal development and reduced drug use for backpackers. Protecting such benefits is in the interest of countries, companies and travellers. However, it requires leadership for collaborative working between nations and partnerships between health and commercial companies, including airlines, holiday companies and Internet sites for backpackers. Such partnerships should deliver information not just on sun-bathing, vaccination and holiday insurance but also on the dangers of substance use. Together they can ensure that the health of backpackers is protected, access to services are adequate and opportunities to provide health information are not missed.

## Competing interests

The author(s) declare that they have no competing interests.

## Authors' contributions

MAB designed and developed the research study, analysed the data and wrote the manuscript. KH developed the research study, input data and assisted in writing the manuscript. PD developed the study, managed the research and edited the manuscript. JC developed the study and edited the manuscript. PG co-ordinated the research project and edited the manuscript. All authors have read and approved the final manuscript.

## Pre-publication history

The pre-publication history for this paper can be accessed here:


